# Spatial variations of conductivity of self-assembled monolayers of dodecanethiol on Au/mica and Au/Si substrates

**DOI:** 10.3762/bjnano.14.97

**Published:** 2023-12-05

**Authors:** Julian Skolaut, Jędrzej Tepper, Federica Galli, Wulf Wulfhekel, Jan M van Ruitenbeek

**Affiliations:** 1 Institute for Quantum Materials and Technology, Karlsruhe Institute of Technology, Hermann-von-Helmholtz-Platz 1, 76344 Eggenstein-Leopoldshafen, Germany; 2 Huygens-Kamerlingh Onnes Laboratorium, Leiden University, Niels Bohrweg 2, 2333 CA Leiden, Netherlands; 3 Physikalisches Institut, Karlsruhe Institute of Technology, Wolfgang-Gaede-Straße 1, 76131, Karlsruhe, Germany

**Keywords:** Au/mica, Au/Si, conductive atomic force microscopy, dodecanethiol, self-assembled monolayers

## Abstract

Determining the conductivity of molecular layers is a crucial step in advancing towards applications in molecular electronics. A common test bed for fundamental investigations on how to acquire this conductivity are alkanethiol layers on gold substrates. A widely used approach in measuring the conductivity of a molecular layer is conductive atomic force microscopy. Using this method, we investigate the influence of a rougher and a flatter gold substrate on the lateral variation of the conductivity. We find that the roughness of the substrate crucially defines this variation. We conclude that it is paramount to adequately choose a gold substrate for investigations on molecular layer conductivity.

## Introduction

For decades, the need for miniaturization of electronics has pushed the research field into the direction of bottom-up, rather than top-down, approaches. In this research field, molecular electronics [1–3] has always held a central role, as the flexibility and control over the structure of molecules is unmatched. One of the fundamental parts of devices employing a bottom-up approach combined with molecular electronics is comprised of metal electrodes and molecular layers deposited onto them.

For the use in applications, the properties of such layers of molecules and the interface they form with the metal substrate have to be investigated carefully and systematically. In order to achieve comparability between different types of molecules, ordered layers are favorable, which makes self-assembled monolayers (SAMs) a perfect test bed for studies on molecular layers.

With the idea of molecular electronics in mind, most studies have been aimed at studying the conductivity of SAMs. In previous studies, the contacting of SAMs has been achieved in various ways [4]. We focus here on the contacting of molecular layers between a metal surface and a locally probing electrode. In early studies using this approach, the layers were contacted by a mercury droplet at the end of an electrode, which was then placed on top of the SAM [5–7]. Applying a voltage and, therefore, a current to the substrate and the mercury electrode yields the conductivity of the SAM, averaged over the contact area of the mercury droplet. In such studies, one of the crucial problems was mercury filling out defects in the SAMs, which leads to short circuits and unreliable currents running through the microcontact.

This was avoided in later experiments by using eutectic GaIn (eGaIn) droplets [8–11]. These are much more viscous, to the point that they are almost solid. This reduces the amount of leak currents significantly and makes studies on the conductivity of SAMs much more reliable. A more widely applied method uses conductive atomic force microscopy (CAFM). In this technique, a conductive probe is used in an AFM, which allows for imaging the surface topography (and other characteristics such as adhesion and stiffness) with lateral resolution while simultaneously being able to measure current characteristics. Moreover, the probes used in CAFM are significantly sharper compared to, for example, mercury droplets or eGaIn, which makes it possible to avoid short circuits to the metallic surface relatively easily.

In previous studies, CAFM has been used to investigate the conductivity of surfaces and SAMs, including many studies performed recently on SAMs of helical oligopeptides studying chiral-induced spin-selectivity [12–15]. Here, we re-examine the information that is obtained from CAFM, and we demonstrate that the nature of the metallic substrate is of critical importance. The lateral variation of current characteristics strongly depends on the substrate chosen to deposit the SAM onto. For this study, we employ alkane thiols, which are allowed to form a SAM on different types of Au substrates. We have chosen dodecanethiol (DDT) molecules and study them on commercially available Au substrates consisting of thin Au layers of different surface roughness. We compare granular Au films deposited on Si wafers with epitaxial (flat) Au films on mica.

## Experimental

Before the experimental results are presented, this section focuses on the preparation of the samples under study and the setup used to carry out the measurements. As mentioned above, two types of Au substrates were used, that is, Au-coated Si (Au/Si) and epitaxially grown Au on mica (Au/mica), bought from Sigma-Aldrich and Phasis, respectively. The Au thicknesses are 200 nm for Au/mica and 100 nm for Au/Si substrates. The Au/mica substrates were used directly out of the box without any further cleaning steps. Au/Si was additionally cleaned by boiling in acetone followed by ethanol for 20 min under a fume hood. It was then dried in a glovebox in N_2_ atmosphere, exposed to ozone to remove organic contaminants, and finally rinsed with warm ethanol.

DDT SAMs were deposited onto these substrates by immersing them in a 10 mM solution of DDT in ethanol with subsequent incubation for 24 h. After transfer into a glovebox, the samples were rinsed with ethanol and dried. To improve the order of the SAMs, they were again immersed in 10 mM DDT/ethanol solution and heated to ≈80 °C for 1 h. After gradual cooldown, the samples were again rinsed and dried in N_2_ atmosphere in the glovebox.

The obtained samples were studied using a commercially available JPK NanoWizard^®^ 3 AFM setup. The setup has been upgraded by a CAFM tip holder with an integrated preamplifier, whose feedback resistor of 1 GΩ fixes the maximum measurable current to 12 nA, sets the amplification to 10^9^ V/A, and allows one to measure currents down to few tens of picoamperes.

In the studies presented here, two types of CAFM-probes were used. For the studies on DDT SAMs on Au/mica, Bruker MESP-V2 (CoCr-coated Si) probes were used, whereas, for the remaining measurements, Rocky Mountain Nanotechnology RMN-25PT300B probes with solid Pt wire as tips were used. The latter have the advantage that they do not oxidize easily and remain conductive, as there is no fragile metal coating on a non-conductive probe in contrast to the CoCr-coated Si probes. This is at the cost of lateral resolution due to the larger radius of the probe apex.

**Figure 1:**
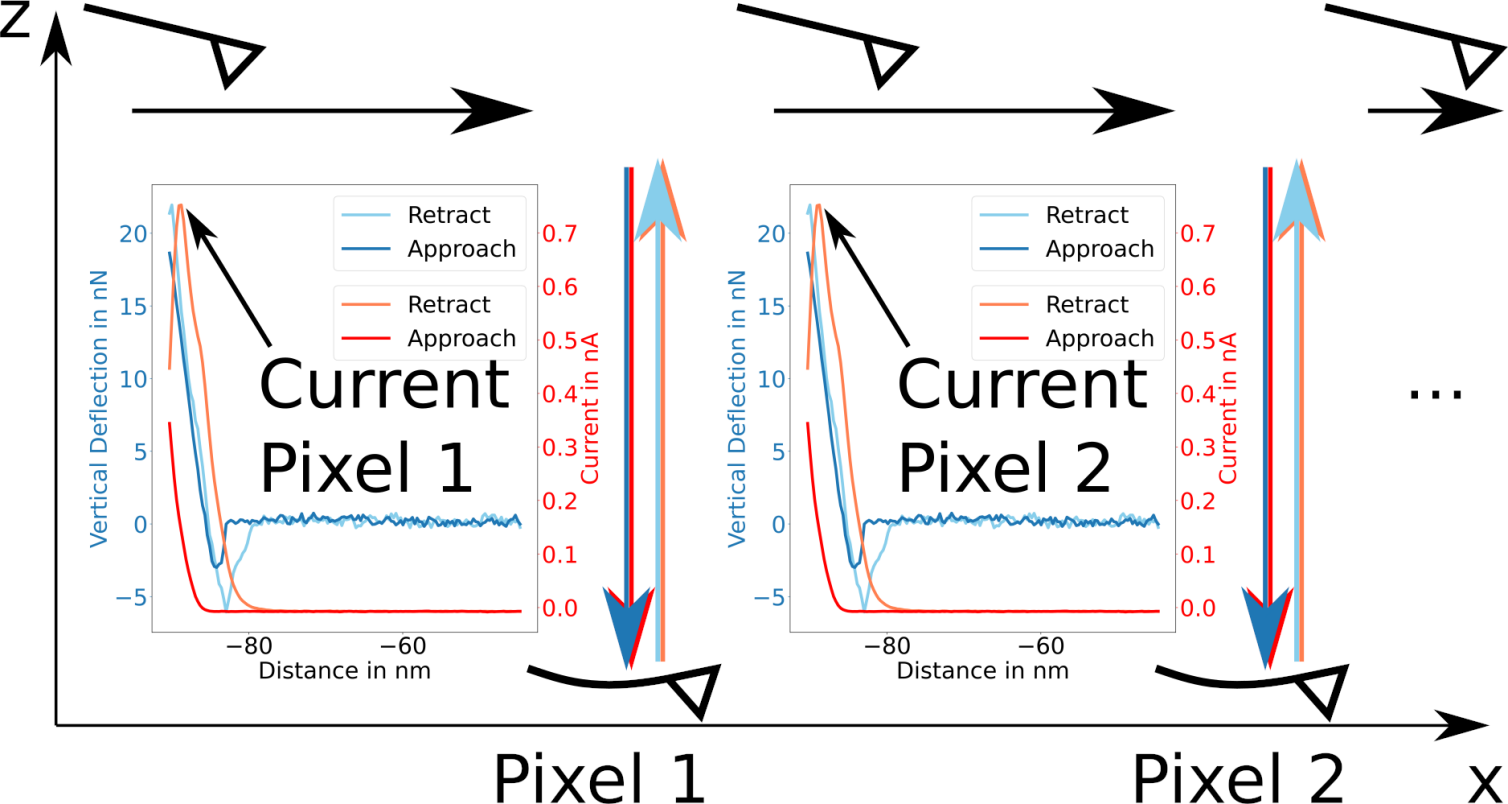
In QI™ mode, the probe measures force and conductance curves at a raster of points on the surface, shown here for a row along the *x* direction. The probe is moved from pixel to pixel in a retracted position far away from the surface. At each point, a force–distance curve is measured while simultaneously acquiring the current. Current maps show the currents extracted as the extremal value of each current curve.

All measurements presented here were carried out in the Quantitative Imaging (QI™) mode by JPK. A sketch of the procedure is shown in Figure 1. In this mode of CAFM operation, a force–distance curve is measured at every pixel of the image. The tip is approached until a certain bend of the cantilever is reached, corresponding to the force setpoint *F*_setpoint_. Plotting the *z* position at which the force setpoint is reached provides the topographic information, which we represent here as a yellow–blue color map. During the whole measurement, a bias voltage *U*_bias_ is applied between tip and sample. Simultaneously to the force–distance curve, the current is acquired. As it can be seen from the example curves in Figure 1, the extremal current is usually found close to the force setpoint, and both are correlated. The small shift of the position of the extremal current towards larger *z* distance can be explained by the bandwidth of the preamplifier (specified as 2 kHz). The relatively high rate of 40 approach/retraction cycles per second was chosen as a compromise between bandwidth distortion and total measurement time.

Plotting the extremal current yields the current maps shown here in gray scale and provides a measure to compare the conductive properties in different areas of the surface. Using the QI mode is particularly advantageous in our study, since it measures topography and current simultaneously and reduces wear effects on the tips.

## Results and Discussion

We divided the results obtained with the methods described above into two main sections. These are studies on (i) the bare substrates and on (ii) the DDT SAMs on these substrates. The bare substrates were investigated as a reference for the measurements thereafter. They show topographies and current maps characteristic for Au/Si and Au/mica. Subsequently, it was observed how these characteristics change with SAMs deposited onto the surface. A strong resemblance between bare and SAM-covered surfaces was observed. This bears important consequences for the choice of substrates for studies on molecular SAMs; flat substrates are advantageous for such studies.

### Bare Au/Si and Au/mica substrates

As mentioned above, two types of Au substrates were investigated, namely Au/Si and Au/mica. The measurements on bare substrates presented here serve as a reference for the studies on the lateral variation of the conductivity of DDT SAMs on said substrates. The reference helps in identifying how much of the SAM’s lateral variation of conductivity stems from the substrate.

**Figure 2:**
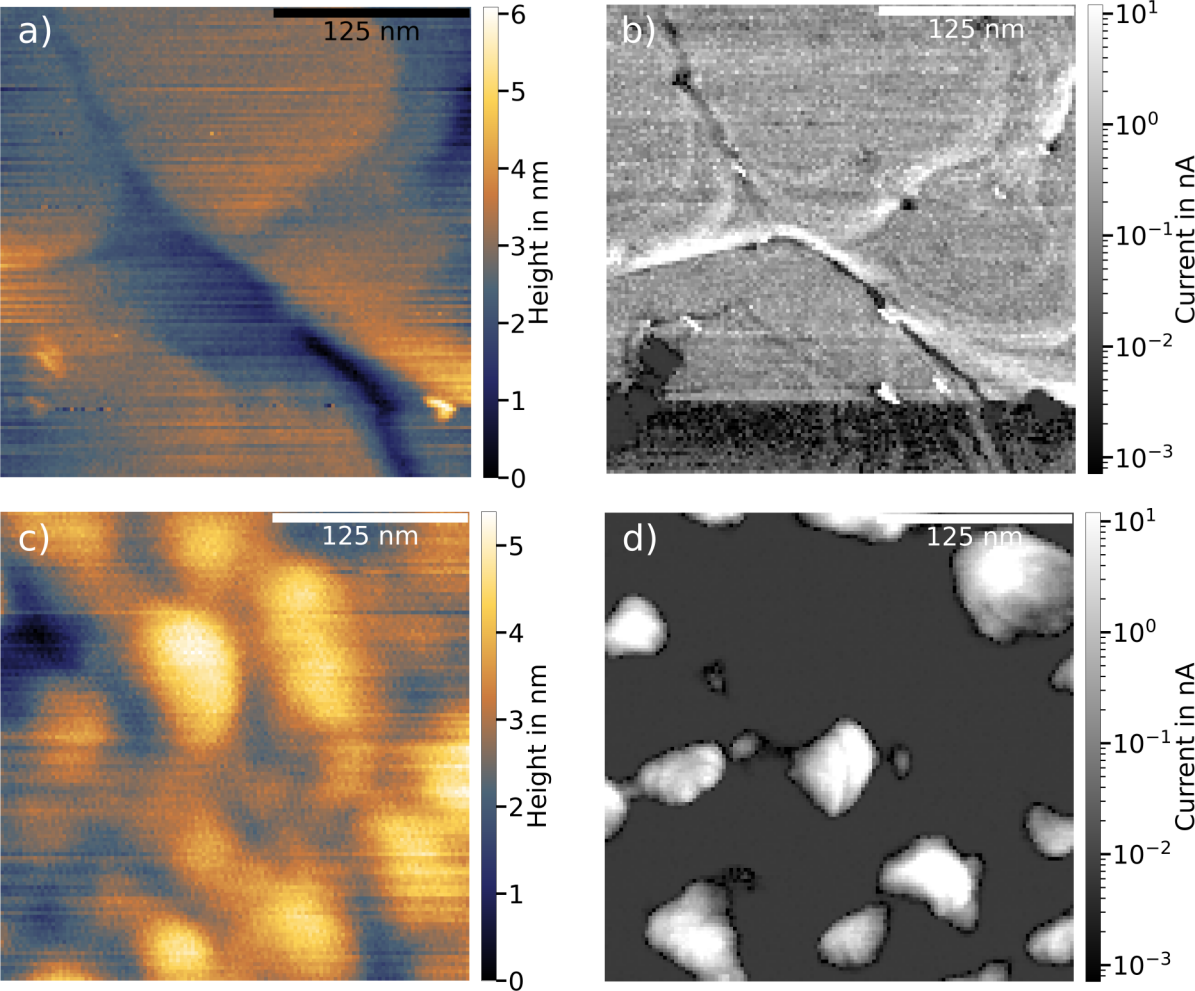
(a, b) Topography and current map, respectively, of the bare Au/mica substrate. The large flat areas provide a homogeneous current distribution throughout large parts of the image. The sudden change in current in the lower part of the image can be attributed to a tip change. (c, d) The same data for a Au/Si substrate. The topography shows more substructures, which is reflected in more extreme current values in the current map (*F*_setpoint_ = 50 nN, *U*_bias_ = 100 mV, RMN probe).

Figure 2a and Figure 2b show the topography and the current map, respectively, for a Au/mica substrate. The 300 × 300 nm^2^ topography map shows that the Au/mica substrate has large flat areas on which the height does not change significantly. The overall change in height throughout the image is approximately 4 nm, and the most significant changes in height occur at the boundaries between different flat areas.

The corresponding current map (Figure 2b) shows a nearly homogeneous distribution of the current throughout the whole image, where the current takes on well-measurable values distributed around 200 pA. Only the edges between the flat areas show significant variation from the homogeneous current distribution, making the terrace edges clearly visible. However, as these edge regions are small compared to the flat areas, the overall current distribution is narrow (see Supporting Information File 1, Figure S2a).

In contrast to the Au/mica surface, the Au/Si substrate exhibits a rougher surface, as seen in Figure 2c, in agreement with the difference in growth mode of Au films on the two substrates. For mica, epitaxial growth is obtained [16–17], while Au on Si/SiO_2_ forms a granular film [18]. Although the overall height variation is not very different from that observed for the Au/mica substrate (approximately 5 nm), the Au/Si surface shows much more substructures and no flat terraces. Compared to the flat terraces of Au/mica, Au/Si has more peaks and valleys, which is also reflected in the current map in Figure 2d. Here, most of the current map is either at the lower limit of the measurable current (few tens of picoamperes) or at the top end of the current range (high-nanoampere regime) (see Supporting Information File 1, Figure S2b). The transition from low to high currents takes place on rather small length scales of tens of nanometers.

The areas of high current appear to coincide with areas of lower topography, slightly skewed to the bottom right of areas with higher topography. This happens all over the image and indicates an effect of the probe influencing the occurrence of high-current areas. The higher currents found in the valleys likely result from varying surface-normal load forces. They are smaller if the probe contacts the surface on a flat area and larger if the contact is on a slope in the topography. As the load force only controls the force component normal to the sample plane, this leads to larger variations in the local normal force when the tip lands on a slope. Therefore, the rougher topography is likely influencing the occurrence of high- and low-current areas. More specifically, this means that the conductance can appear higher on slopes and rough surfaces, as the tip contacts the surface laterally.

This rationalizes the large systematic difference between the two substrates regarding their topography and lateral current variation. The Au/mica substrate shows a flatter topography accompanied by a more homogeneous current distribution. Generally, this is favorable for current measurements on SAMs, as it provides larger areas of comparable current to study the conductive properties of molecular SAMs and their lateral variation. With the lateral variation of the current of the bare substrates being known, a well-founded description of the changes after SAM deposition can be made.

Overall, five areas were investigated on Au/mica and three for Au/Si, which all showed consistent images.

### Dodecanethiol SAMs on Au/mica

**Figure 3:**
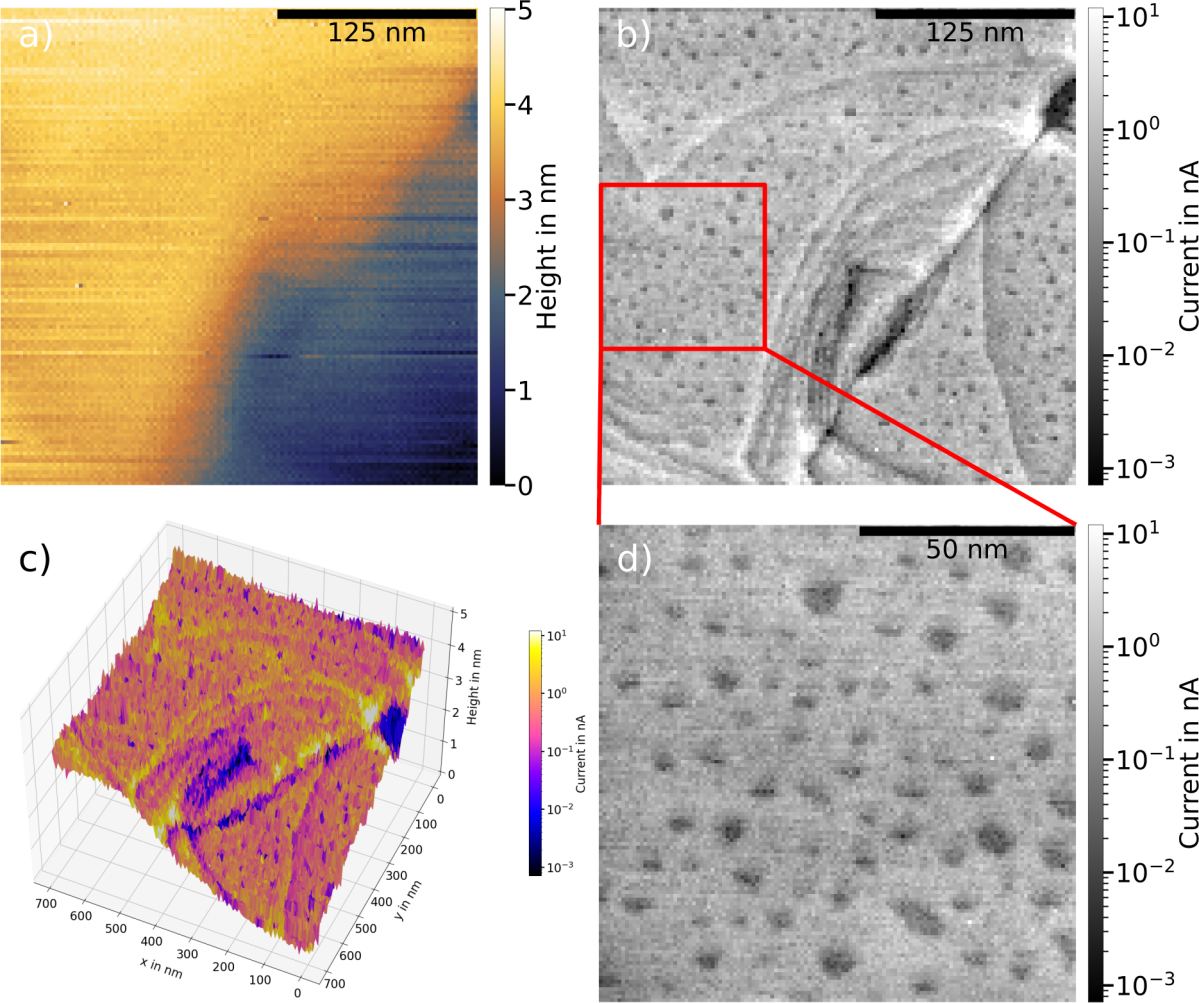
(a, b) Topography and current map, respectively, for a DDT SAM on Au/mica, obtained simultaneously on the same area. The current map reveals additional features of the substrate such as shallow terraces and etch pits. The closeup in (d) shows that the etch pit size matches the expected value of a few nanometers. (c) 3D view of the surface, in which the color coding indicates the measured current at each point, clarifying the connection between topography and current. The point of view is adjusted for best visibility (*F*_setpoint_ = 80 nN, *U*_bias_ = 1 V, MESP probe).

Figure 3 shows images of the DDT-covered Au/mica surface obtained after the deposition procedure described above. In total, four different areas on two DDT/Au/mica samples were investigated, yielding consistent results. In Figure 3a, the topography is similar to that obtained for the bare Au/mica surface, that is, relatively large flat areas, only small height differences throughout the image, and small roughness of the surface. By means of topography alone, the surface cannot be distinguished from that of the bare Au/mica surface.

Also, upon looking at the current distribution, many features are similar to those of the bare Au/mica surface, including its homogeneous current distribution, for this specific case (Figure 3) around 800 pA. It is sensible that the average current is higher compared to the measurements on the bare Au/mica substrate (Figure 2b), as force setpoint and bias voltage are higher. The average current value of 800 pA is again well measurable and allows for a rough estimation of the resistance of each molecule. Such an estimation can be done without consideration of the resistance of the bare Au substrate, as its resistance is much lower than the SAM’s resistance. Assuming that approximately 1000 molecules are contacted [4] and all contacted molecules are connected in parallel, the total resistance of *R*_tot_ = *U*_bias_/*I* = 1.25 × 10^3^ MΩ results in a resistance for one molecule of *R*_mol_ = 1.25 × 10^6^ MΩ. Comparing this value to the literature value ranging between 10^6^ and 10^7^ MΩ per molecule, as presented in [4], shows reasonable agreement.

In addition to the features observed for the bare substrate, including its homogeneous distribution around well-measurable current values, more features are visible in the current map (Figure 3b). First, finer topographic details, namely shallower terraces and boundaries between flat areas of the topography, possibly step edges between single atomic steps of the Au surface, become clearly observable. Second, dark spots on the terraces of the current map appear, which can be seen clearly in the expanded-scale image in Figure 3d. These can be attributed to so-called etch pits that arise from the growth of sulfur-bound SAMs on Au surfaces [19–20]. These etch pits are monatomically deep holes in the Au surface. They are produced in the process of SAM formation by sulfur–gold bonds, which result in removing Au atoms from the top layer. This leaves the surface with Au atom vacancies that arrange into small islands of a few nanometers in size. This size matches the darker areas observed in the current maps after DDT SAM formation. The presence of a well-ordered SAM on the surface was confirmed by scanning tunneling microscopy (STM) images on alkanethiol-covered Au surfaces prepared in the same way, in which the individual molecules can be resolved, shown in Figure S4 in Supporting Information File 1. The etch pits serve as evidence that the SAMs form in an ordered fashion. The abovementioned features can also be seen clearly in the 3D view of the surface in Figure 3c, where the color coding indicates the measured current at each point. The 3D view also underlines the direct correspondence between features in the current map and the topography.

A further indication that the SAM has formed correctly is the observation that it can be thinned by imaging smaller areas with high load forces. As shown in Figure S5 in Supporting Information File 1, after three consecutive imaging runs performed on the same area, the center square of the image appears lower in topography compared to the sides when the scanning area is widened. Also, the measured current increases from image to image, while the etch pits remain intact, indicating that the Au surface structure remains unaffected. We attribute lower topography and increased current to a thinning of the SAM by pushing aside molecules with the probe. Another effect contributing to the thinning of the SAM is molecules being picked up by the probe during the measurement. The effect we observe here is most likely a combination of both processes.

All these indications lead to the conclusion that ordered DDT SAMs form on the surface with the chosen deposition technique. More importantly, the current maps in Figure 3 show that substrate and measurement technique are suitable for obtaining information on the conductivity of a molecular SAM, as the measured currents show a homogeneous distribution and large areas without change in the topography, allowing for comparison between the currents measured on these areas. For quantitative information, it is also important to reduce the load force as suggested by the observed removal of part of the SAM by the tip during imaging. Studies of the extent and type presented here can be used as the basis for well-founded statements concerning electronic properties such as the current–voltage characteristics of the molecular SAM. To this purpose, the characteristics should only be averaged over comparable areas, excluding terrace boundaries and other edges. As the Au/mica substrates provide large areas of this kind, they are favored for the use in studies of the conductive properties of SAMs.

### Dodecanethiol SAMs on Au/Si

The SAM formation technique used for the Au/mica substrates was also used for the Au/Si substrates. As it seems to be suitable for Au/mica substrates, it should also yield densely packed molecular SAMs on Au/Si substrates, allowing for the evaluation of the influence of the substrate on the lateral variation of the conductive properties of SAMs.

**Figure 4:**
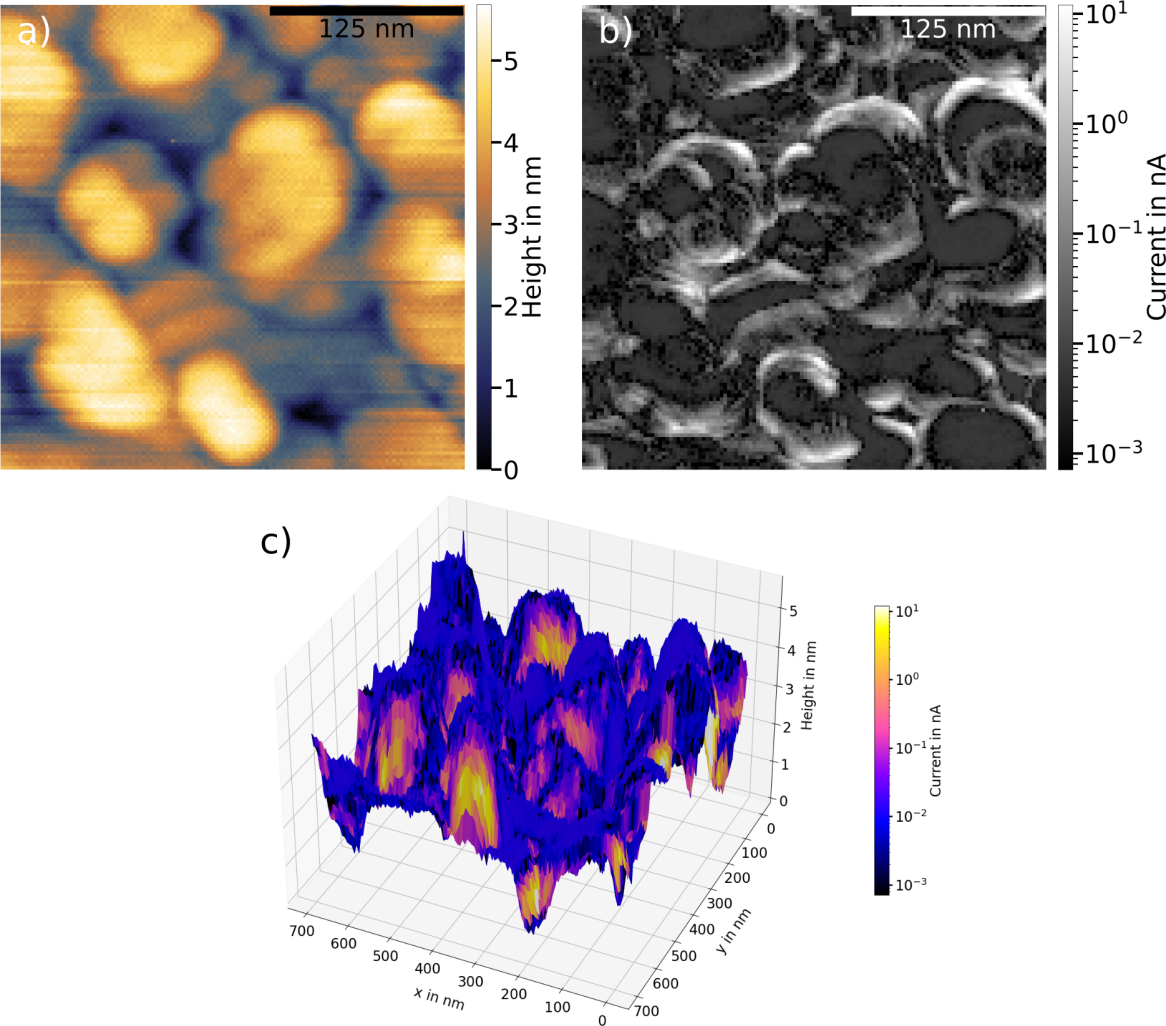
(a, b) Topography and current map, respectively, of a DDT SAM on a Au/Si substrate. The rougher surface seen for the bare substrate is also found here together with its influence on the current map. Very small currents dominate the map, changing to high currents rather abruptly. This yields only small areas with measurable currents, which is unfavorable for the averaging of conductive properties of the surface. The 3D view in (c) emphasizes the correlation between the rougher surface structure and the high currents on the slopes of the grains. The point of view is adjusted for best visibility (*F*_setpoint_ = 20 nN, *U*_bias_ = 1 V, RMN probe).

For comparison, Figure 4 presents measurements of DDT SAMs on a Au/Si substrate. Comparing topography (Figure 4a) and current map (Figure 4b) to the ones of the bare Au/Si substrate, close similarities can be seen. After coverage of the surface by the SAM, the surface retains the same roughness with only small flat areas. Although in Figure 4 this is slightly distorted by a probe effect duplicating features, the systematic difference in surface structure between Au/Si and Au/mica, already observed in the bare substrates, is reproduced.

Just as for the bare Au/Si, the substructures of the substrate lead to strong variations in the corresponding current map. There are large areas with very small currents on the flatter areas of the topography. The current rather abruptly increases at the slopes of the topography. The 3D view of the surface in Figure 4c, represented in the same way as in Figure 3c, shows clearly that large currents can only be observed at the slopes of the topography as is also the case for bare Au/Si. The flatter areas, however, show very low current values, close to the lower limit of observability. The 3D view also emphasizes the higher roughness of the surface of the DDT SAM on Au/Si compared to the Au/mica substrate.

Additional measurements on SAMs of sulfur-bound oligopeptides (SH–(CH_2_)_2_NH–(Ala-Aib)_5_–COOH) [12] on Au/mica substrates yielded no measurable currents and are therefore omitted in this report.

Our observations show that, when studying the conductive properties of DDT SAMs on Au/Si, the variation in the current is governed by the structure of the substrate, which remains qualitatively unchanged by the deposition of the SAM. For the Au/Si substrate, the rough topography yields only small areas on the surface on which comparable conductive properties can be expected. Without information on the surface topography, the conductance obtained from averaging over random points on the surface [12,21–24] is prone to incorrect averaging. The lateral variation of the conductive properties limits strongly the amount of lateral probe positions over which measurements of such characteristics can be averaged. Using the Au/mica substrate, however, yields large areas of comparable conductive properties, which makes it more suitable for such investigations. Moreover, the strong lateral variation in the current map of Au/Si suggests that it is necessary to choose the points for averaging carefully. A suitable way to do so would be through imaging the surface as presented here.

## Conclusion

This report shows that the lateral variation of the conductive properties of molecular SAMs is governed by the choice of the substrate. To achieve comparable, well-measurable currents and conductive properties, flat substrates are favorable. The flatness of the substrate and homogeneity of the current distribution with and without the SAM should be studied in advance to ensure comparability. A rougher substrate surface leads to stronger variations in the conductive properties, limiting the areas over which conductive properties can be sensibly averaged, and should therefore be avoided.

Moreover, the studies presented here show, that a careful study of the correlation between topography and conductive properties of SAMs is strongly advised, especially if CAFM is used to perform the characterization of the conductive properties of the SAM. With such combined investigations, the areas for averaging can be chosen in a sensible way to reproducibly characterize the SAM’s conductivity, for example, by using only the flat areas of the surface and excluding areas with large slopes in the topography.

## Supporting Information

File 1 Additional figures.
