# Supplementary material for: Spatial variations of conductivity of self-assembled monolayers of dodecanethiol on Au/mica and Au/Si substrates
Source: Beilstein J Nanotechnol. 2023 Dec 5;14:1169–77. doi: 10.3762/bjnano.14.97 (PMC10714468; doi:10.3762/bjnano.14.97)
Supplement: File 1 — Additional figures. [file Beilstein_J_Nanotechnol-14-1169-s001.pdf]

BEILSTEIN JOURNAL OF NANOTECHNOLOGY

Supporting Information

for

Spatial variations of conductivity of self-assembled monolayers of dodecanethiol on Au/mica and Au/Si substrates

Julian Skolaut, Jędrzej Tepper, Federica Galli, Wulf Wulfhekel
and Jan M. van Ruitenbeek

Beilstein J. Nanotechnol. **2023,** *14,* 1169–1177. doi:10.3762/bjnano.14.97

Additional figures

License and Terms: This is a supporting information file under the terms of the Creative Commons Attribution License (https://creativecommons.org/licenses/by/4.0). Please note that the reuse, redistribution and reproduction in particular requires that the author(s) and source are credited and that individual graphics may be subject to special legal provisions.
The license is subject to the *Beilstein Journal of Nanotechnology* terms and conditions: (https://www.beilstein-journals.org/bjnano/terms)

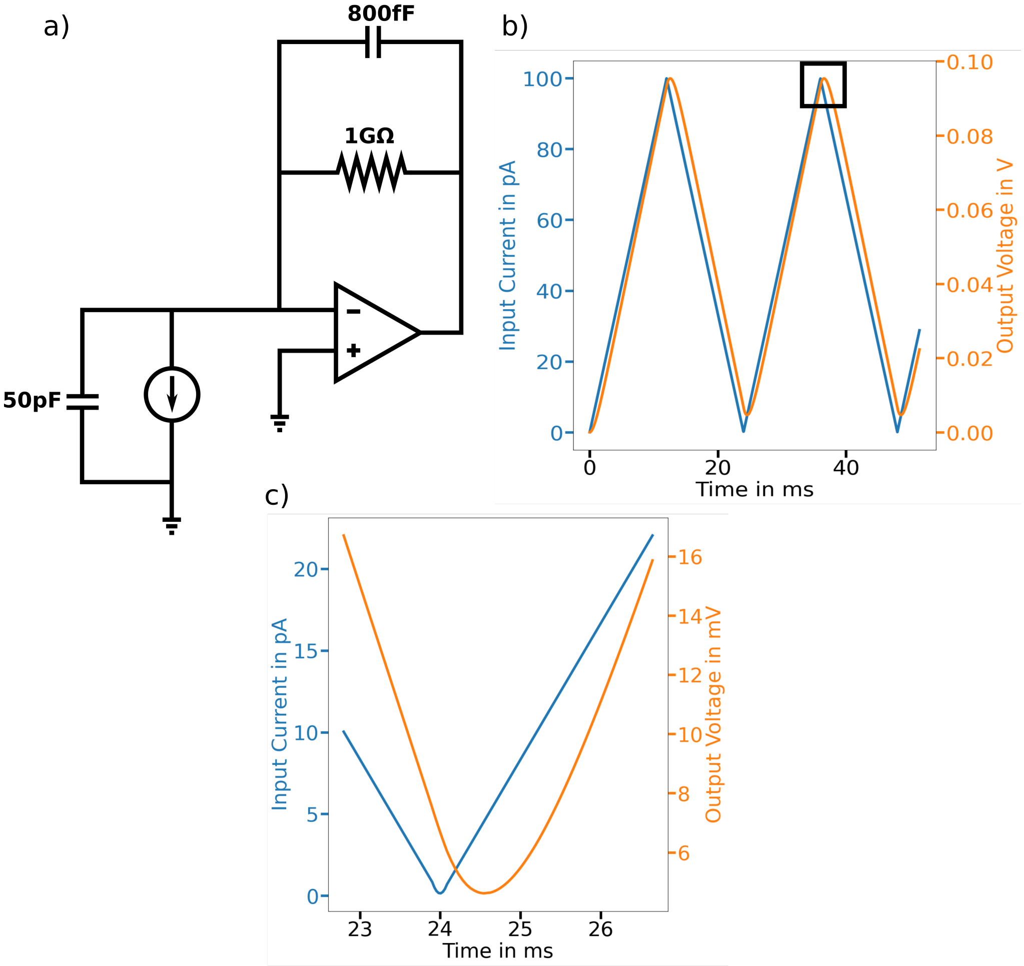

Figure S1: Simulation of the response of a transimpedance amplifier to a zigzag current signal, similar to the one received in the measurements shown in the main text. a) The modeled transimpedance amplifier circuit with parasitic capacitances of the current source and feedback resistor. The current source provides a linearly increasing current with an amplitude of 100 pA and a period of 24 ms, reflecting the time of one full approach-retract cycle of a typical QI™mode measurement. The output voltage is picked up at the output of the operational amplifier. b) The output voltage reflects the input current with the expected amplification of 10^9 with slight distortions. c) Zoom of the signals marked with a black box in b). The output signal is shifted by approximately 0.5 ms with respect to the input signal. Such a shift can cause the artifact of the extremal current value always being in the retract curve of the I–z curve at one pixel of a QI™mode measurement.

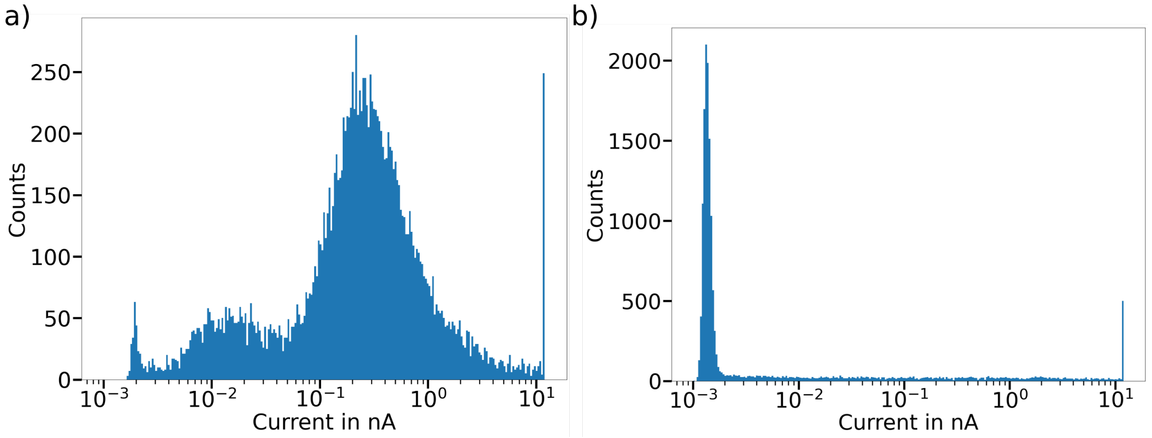

Figure S2: a) shows the current distribution of the bare Au/mica surface shown in the main text. It exhibits a well-defined distribution of currents around a central current of approximately 200 pA. The smaller peak can be attributed to the lower part of the image, where the tip changed, which in turn changed the measurable current. The current distributions for the other samples on the Au/mica substrate are similar in their distribution, usually lacking the second peak feature and the center current varies. The large narrow peak at high currents is an artifact due to the maximum of the measurable current range. b) shows the current distribution of the bare Au/Si surface. The two visible features are peaks at the extremal values of the measurement range of the setup. No clear peak feature can be seen at well-measurable currents.

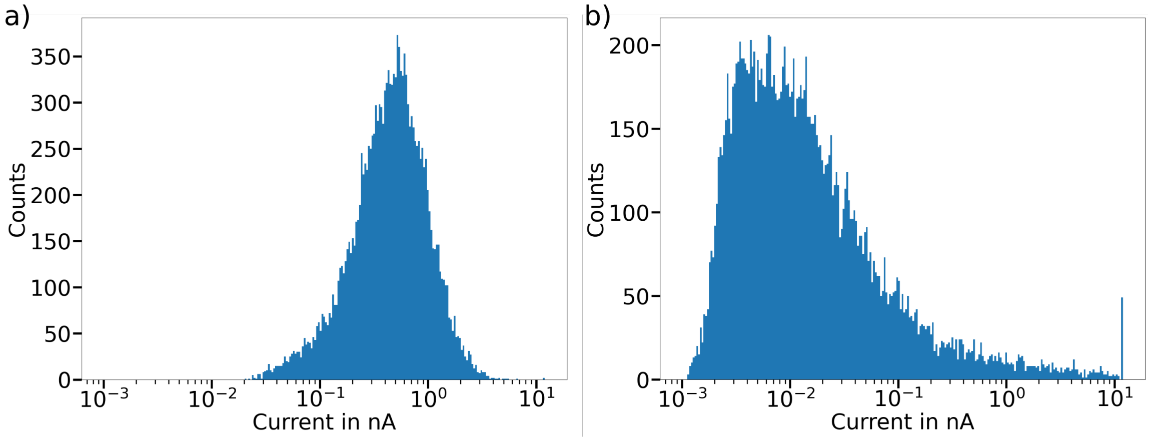

Figure S3: a) shows the current distribution of Figure 3b) (DDT/Au/mica) in the main text. A comparably narrow distribution of well-measurable currents can be seen. b) shows the current distribution for Figure 4b) (DDT/Au/Si) in the main text. It exhibits a wider distribution of currents.

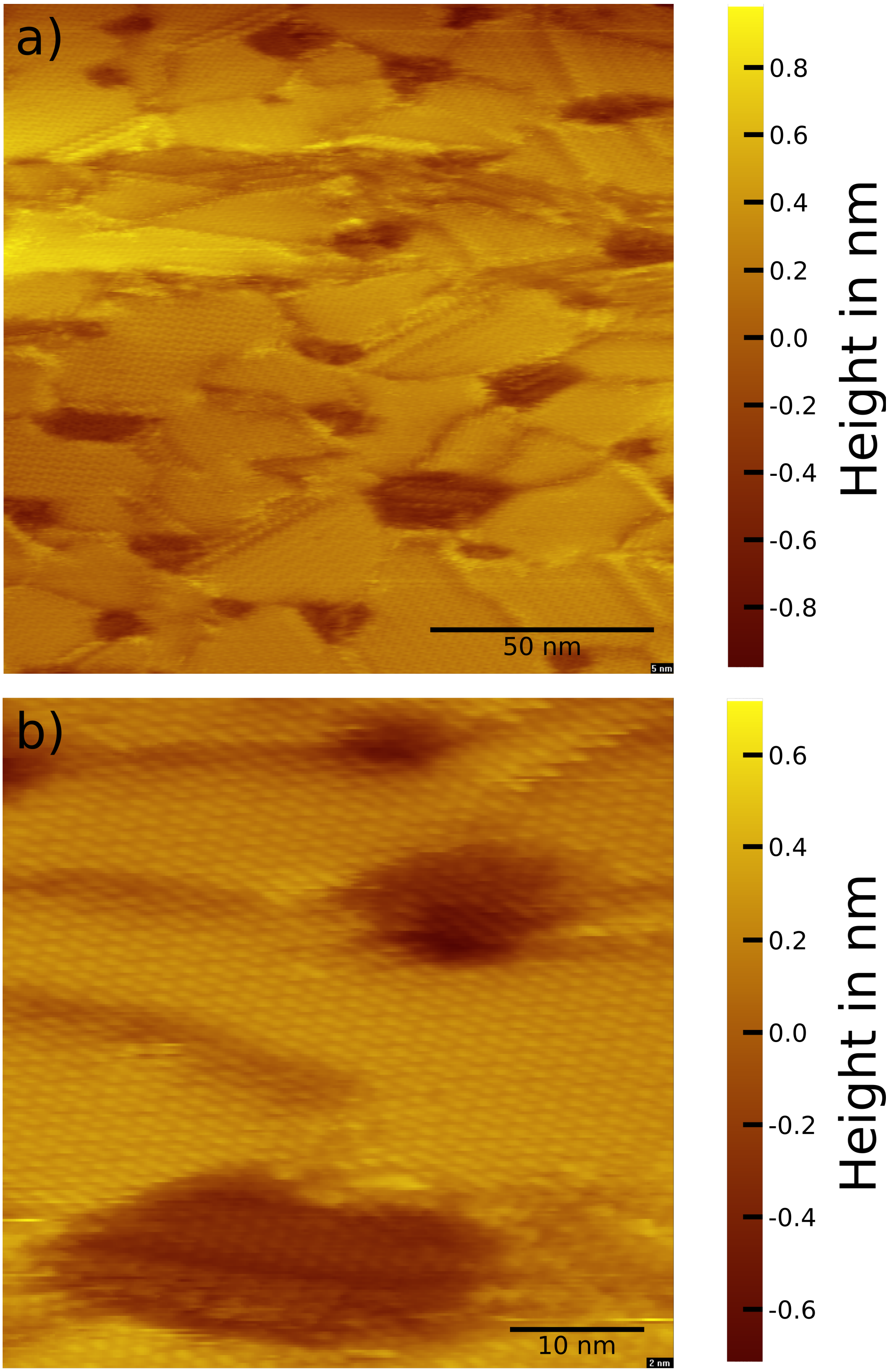

Figure S4: Scanning Tunneling Microscopy (STM) images of a self-assembled monolayer of DDT on Au/mica. The wide area view in a) covers 150 × 150 nm and shows many flat terraces covered with a fine regular pattern due to the DDT molecules. This fine pattern is more clearly visible in the 50 × 50 nm image of panel b). The images were recorded under ambient conditions, and at bias and current set points of 1.1 V and 5 pA in (a), and 0.9 V and 1 pA in (b).

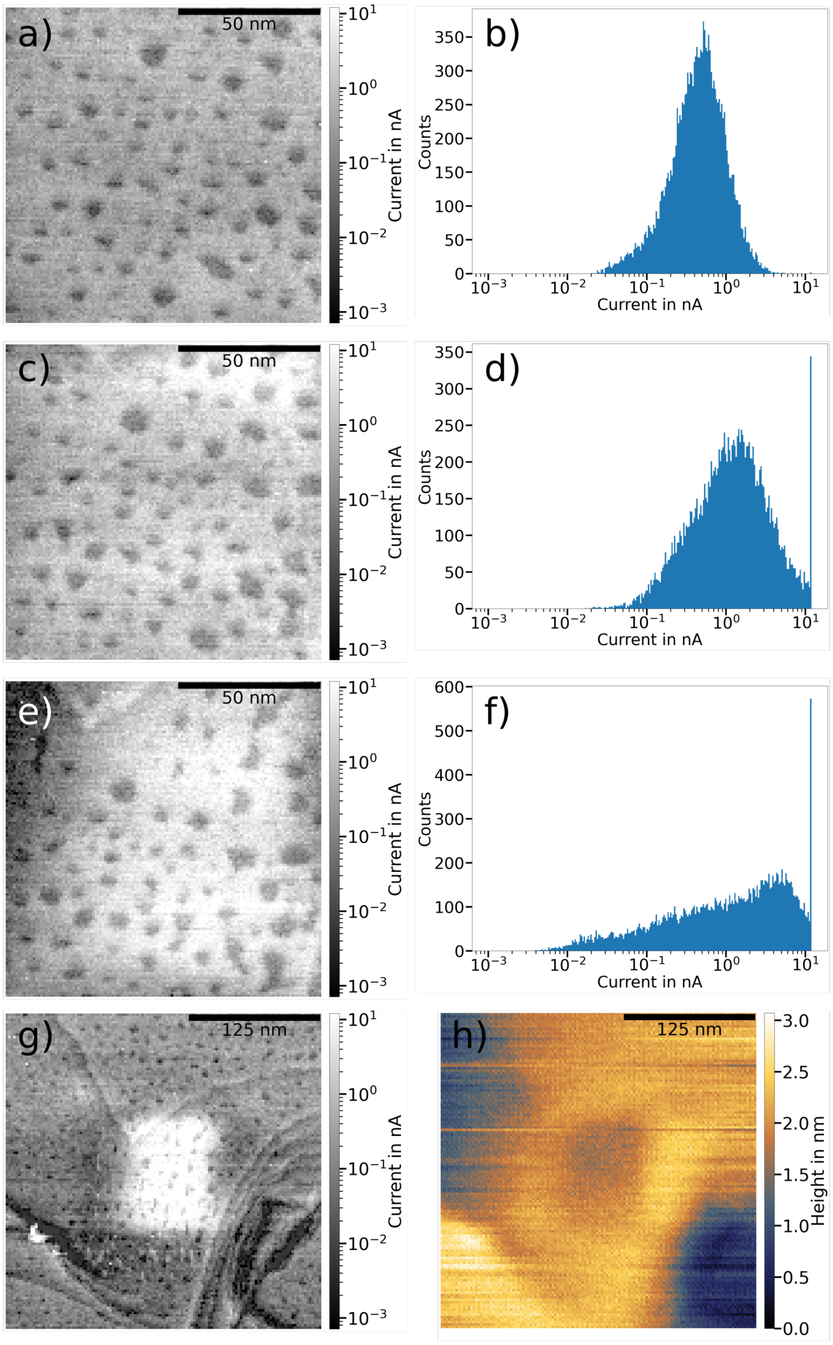

Figure S5: a), c) and e) show the current maps of the same area of the DDT/Au/mica surface indicated in Fig. 3d) in the main text. It becomes apparent black that the current, notably in the center of the images, increases between subsequent images. This is due to the high applied load force of 80 nN, which is enough to at least partially remove the SAM. However, the areas of lower current corresponding to the etch pits (and therefore the Au surface) stay intact. b), d), and f) show the corresponding current distributions, which clarify directly, that the current distribution is shifted to higher currents. g) and h) present the current map and topography of a zoom-out of the same area. The area of removed SAM is clearly visible, as an indentation in the topography and an area of higher current in the current map (F_{setpoint} = 80 nN, U_{bias} = 1 V, MESP probe).

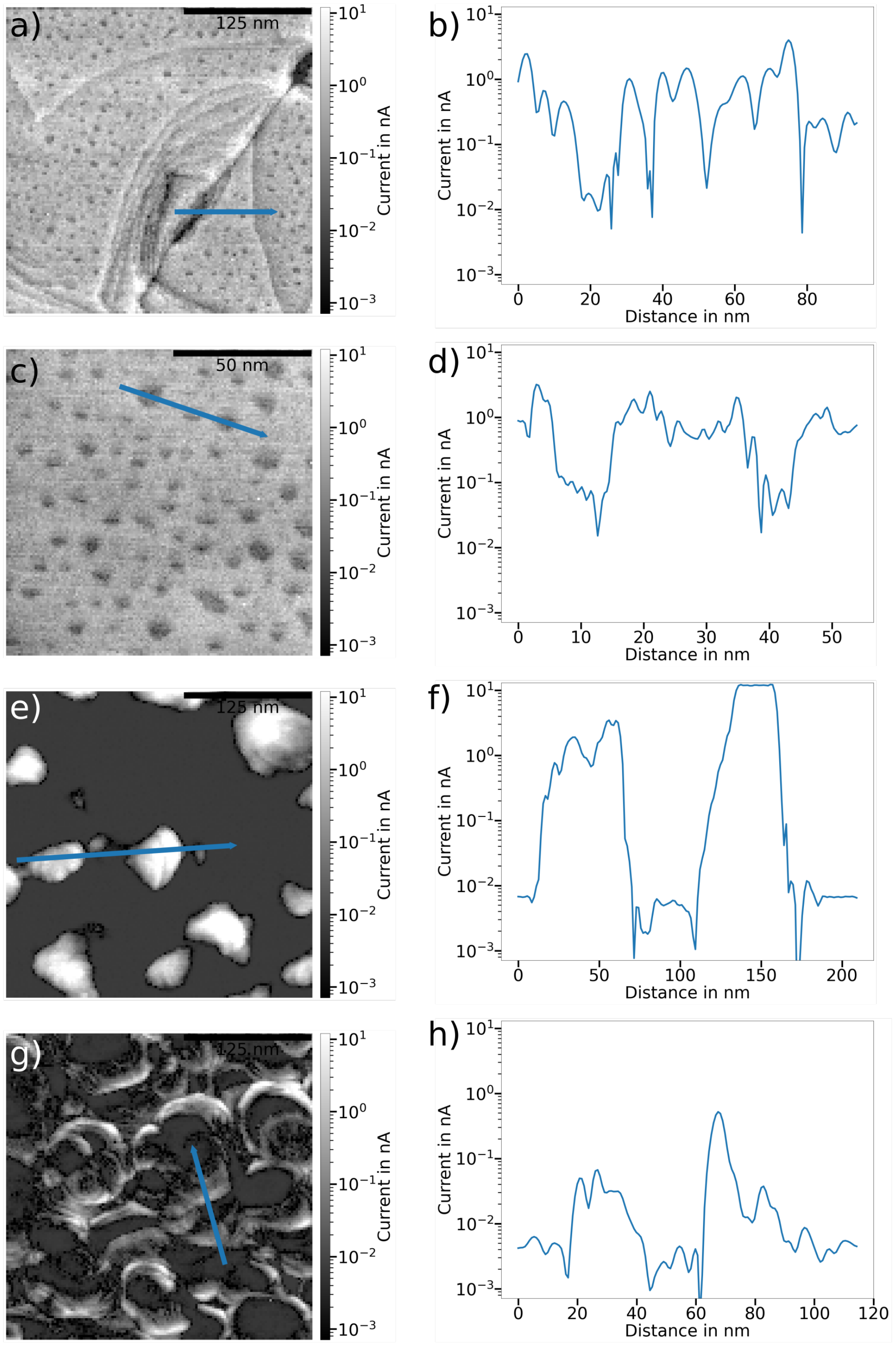

Figure S6: Linecuts across the current maps shown in the main manuscript and Figure S5. The left column (a,c,e,g) show current maps and an arrow indicating of the linecut. The right column (b,d,f,h) show the corresponding linecuts using the same limits and logarithmic scaling in current. These linecuts help to see the differences in current and the variation across the maps.
